# A cross-sectional assessment of the quality and reliability of sleep disorder information on Douyin (TikTok in China)

**DOI:** 10.1038/s41598-026-54965-4

**Published:** 2026-06-03

**Authors:** Yanbin Lin, Hongli Tao, Guoying Sun, Shanshan Zhao, Lina Wang

**Affiliations:** Department of Psychiatry, Xianyang Central Hospital, No. 78, East Renmin Road, Xianyang, 712000 China

**Keywords:** Sleep wake disorders, Social media, Health communication, Video recording, Information quality, Cross-sectional studies, Health literacy, Health care, Medical research

## Abstract

Short-video platforms such as TikTok have become significant sources of health information, particularly among younger populations. However, the quality and reliability of health-related content on these platforms remain a concern. This investigation examines the current landscape of sleep disorder information on Douyin (TikTok in China). To evaluate the quality, trustworthiness, and thematic completeness of sleep disorder-related short videos on Douyin (TikTok in China). We conducted a cross-sectional study by retrieving the top 200 videos from Douyin using a combination of three Chinese keywords (“睡眠障碍” [sleep disorders], “失眠” [insomnia], and“睡眠” [sleep]). The top 200 videos from each keyword search were pooled. After removing duplicate listings, commercial advertisements, and off-topic videos, 154 videos were included in our final study. Engagement metrics—including likes, comments, favorites, and shares—were collected to quantify the influence of the short videos. The quality of these videos was evaluated using the modified DISCERN instrument, the JAMA benchmark criteria, the Global Quality Scale (GQS), and a content-specific tool designed to assess six key domains. Double-blinded assessments were performed. Most videos were created by healthcare professionals. Overall quality was moderate (median scores: GQS = 2, JAMA = 2, mDISCERN = 2). Notable content gaps were identified: 34.4% lacked information on detection, 40.3% provided minimal treatment details, and 52.6% omitted outcomes. Videos from healthcare professionals scored significantly higher (*p* < 0.05). Engagement metrics correlated positively with quality scores. Sleep disorder-related content on Douyin (TikTok in China) is of moderate quality, with professional sources demonstrating superior reliability, suggesting the need for greater efforts to standardize health-related content on such platforms. Strengthening content verification and encouraging professional involvement are essential.

**Supplementary Information:**

The online version contains supplementary material available at 10.1038/s41598-026-54965-4.

## Introduction

Sleep disorders represent a significant and growing public health challenge worldwide, adversely affecting the physical, psychological, and social well-being of millions of individuals^1^. Nowadays, the rapid global ascent of short-video social media platforms has transformed the public’s engagement with health-related information^2^. These platforms combine a highly visual and engaging format with algorithmic distribution, making them particularly appealing to younger demographics as an important source for informal health advice, peer support, and shared experiences.

On Douyin, the Chinese domestic counterpart of TikTok, repositories of content tagged with hashtags like #sleepdisorder and #insomnia have emerged. This phenomenon offers a unique opportunity to raise public awareness about sleep health, reduce stigma, and foster supportive communities^3^. However, the very features that drive the platform’s popularity its emphasis on entertainment and viral potential also pose considerable risks. The competition for user attention can incentivize creators to prioritize emotional appeal and shareability over scientific rigor, sometimes at the expense of accuracy^4,5^.

While the current study does not directly measure downstream clinical outcomes, existing health communication literature suggests that the dissemination of unverified remedies or highly commercialized medical content on short-form platforms can theoretically lead to detrimental real-world behaviors. These background risks include delayed medical consultations, heightened patient anxiety, or the inappropriate self-adoption of unproven self-management practices. Consequently, evaluating the objective quality, reliability, and completeness of sleep-related information within this specific digital environment is critical to understanding and mitigating these public health communication dynamics^6^.

Despite Douyin’s influential role in health communication, systematic evaluations of content quality within specific medical domains on the platform remain limited. While previous studies have examined topics such as mental health and vaccination^7,8^, a critical gap exists in the rigorous assessment of videos pertaining to sleep disorders^9^. This study aims to evaluate the quality, source reliability, and content comprehensiveness of sleep disorder-related short videos on Douyin (TikTok in China), and to discuss its implications for public health communication^10^.

## Methods

### Data collection and screening

A cross-sectional analysis was performed on publicly available Douyin (TikTok in China) videos from September 9–11^11^, 2025. To minimize personalized recommendation bias, data acquisition was conducted using a desktop web browser (Google Chrome, version 120.0) running on a Windows 11 operating system with no user login. The web browser’s location and language settings were set to Mainland China/Simplified Chinese to align with Douyin’s primary target environment.

We searched the platform using Chinese keywords for sleep disorders and selected the top videos. Three separate searches were performed using the default ranking algorithm with the Chinese keywords:“睡眠障碍” (sleep disorders), “失眠” (insomnia), and “睡眠” (sleep). The top 200 videos retrieved from each keyword search were pooled together, yielding an initial pool of 600 video listings. A screening process was then executed to filter duplicates (e.g., cross-listed videos across the three keywords), off-topic videos, and clear commercial advertisements. This systematic screening resulted in a final analytical sample of 154 unique videos.

### Variable extraction and classification

For each included video, researchers documented eight key parameters: video title, four engagement metrics (likes, comments, favorites, shares), duration, and source classification^12^. Both visual and auditory content were reviewed; no formal transcript analysis was performed. The engagement metrics—including likes, comments, favorites, and shares—were used to quantify the influence of the short videos. Content creators were categorized based on platform-verified information into four mutually exclusive groups: psychiatrists, other specialist physicians, news agencies, and individual users. “Platform-verified” status was defined by the presence of Douyin’s official verification badges. For healthcare professionals, verification required official platform-certified medical registration credentials (e.g., hospital employment and clinical practicing licenses) visible on their profile. News agencies were verified via official media enterprise credentials. Individual users consisted of accounts without professional medical or media verification badges. Any ambiguous cases were reviewed collectively by the research team and classified based on the account’s primary published content history or excluded if identity could not be verified. These categories were subsequently consolidated into two broader groups: healthcare professionals (psychiatrists and other specialists) and non-healthcare professionals (news agencies and individual users).

### Quality assessment protocol

Video quality and reliability were evaluated using three adapted instruments: the modified DISCERN tool, JAMA benchmark criteria, and Global Quality Scale (GQS)^13–15^. The modified DISCERN instrument examined compliance with five criteria: clarity, relevance, traceability, robustness, and impartiality, with binary scoring (yes = 1, no = 0) producing a total score of 0–5. The JAMA benchmark evaluated four dimensions authorship, currency, validity, and conflict disclosure generating composite scores from 0 to 4. The GQS employed a 5-point Likert scale (1 = very poor to 5 = very good) to assess overall quality based on professionalism, comprehensiveness, clarity, and understandability.

Content comprehensiveness was measured using a specialized tool evaluating six domains: definition, symptoms, risk factors, detection, treatment, and outcomes^16^. Each domain received tiered scoring.

To ensure assessment reliability, a double-blinded evaluation process was implemented where reviewers independently scored videos without accessing each other’s ratings^17^. Two reviewers with training in public health independently scored all videos. Inter-rater reliability was substantial, with Cohen’s kappa values of 0.78 for mDISCERN, 0.75 for JAMA, and 0.73 for GQS. A third reviewer was consulted for 18 videos (11.7% of the sample) to resolve scoring discrepancies. Discrepancies were resolved through third-reviewer consultation. All evaluators completed standardized training on assessment criteria prior to data collection to enhance scoring consistency.

### Statistical analysis

Data analysis utilized IBM SPSS Statistics (Version 26.0). Non-normally distributed continuous variables (such as video duration and engagement metrics) were analyzed using the Kruskal-Wallis H test for comparisons across the four creator groups and reported as median with interquartile range (IQR). For comparisons between two consolidated groups (healthcare professionals vs. non-healthcare professionals), the Wilcoxon rank-sum (Mann-Whitney U) test was applied. Normally distributed variables underwent independent samples t-tests. Statistical significance was established at *p* < 0.05.

**Table 1:** Video characteristics.

Characteristic	Value (*N* = 154)
Video source, n (%)	
Psychiatrist	44(28.6)
Other specialist physicians	65(42.2)
News agencies	27(17.5)
Individual users	18(11.7)
Video duration [s, median (IQR)]	41(24-86.25)
Number of likes [median (IQR)]	447(66-3967.75)
Number of comments [median (IQR)]	31(5-249.75)
Number of collections [median (IQR)	122(19-1405)
Number of shares [median (IQR)]	129(13.75-1496.75)
GQS scores [median (IQR)]	2(2–3)
modified DISCERN scores [median (IQR)]	2(2–3)
JAMA scores [median (IQR)]	2(1–3)

GQS: Global Quality Score; IQR: interquartile range;

**Table 2:** Completeness of video content.

Video content	Definition (*n* %)	Sign/symptoms (*n* %)	Risk factors (*n* %)	Test (*n* %)	Treatment management (*n* %)	Outcomes (*n* %)
No content (0 point)	24 (15.6)	0 (0%)	11 (7.1)	53 (34.4)	29 (18.8)	22 (14.3)
Little content (0.5 point)	58 (37.7)	36 (23.4)	44 (28.6)	59 (38.3)	62 (40.3)	59 (38.3)
Some content (1 point)	57 (37.0)	38 (24.7)	39 (25.3)	38 (24.7)	58 (37.7)	61 (39.6)
Most content (1.5 point)	12 (7.8)	26 (16.9)	9 (5.8)	4 (2.6)	4 (2.6)	11 (7.1)
Extensive content (2 point)	3 (1.9)	54 (35.1)	51 (33.1)	0 (0)	1 (0.6)	1 (0.6)

## Results

### Selection of short videos

**Fig. 1:**
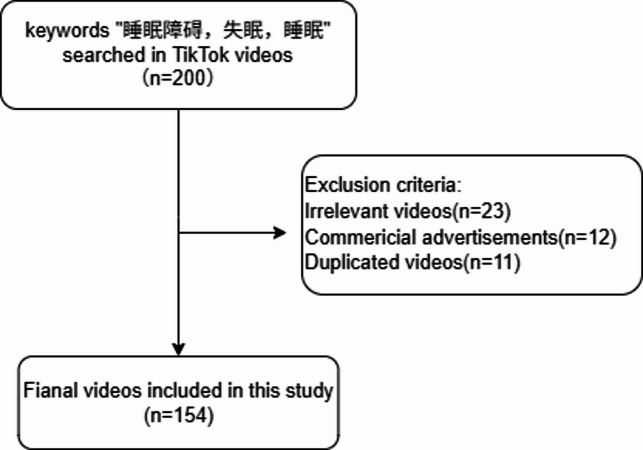
The flow chart of this study.

The video selection process commenced with the top 200 videos by comprehensive rank on Douyin. Upon applying the eligibility criteria, 46 videos were excluded. Following the exclusion of 11 duplicates, 23 theme-irrelevant videos, and 12 commercial advertisements, a total of 154 videos qualified for inclusion in the study. A detailed flowchart of this process is provided in Fig. 1.

### Short videos characteristics

**Fig. 2:**
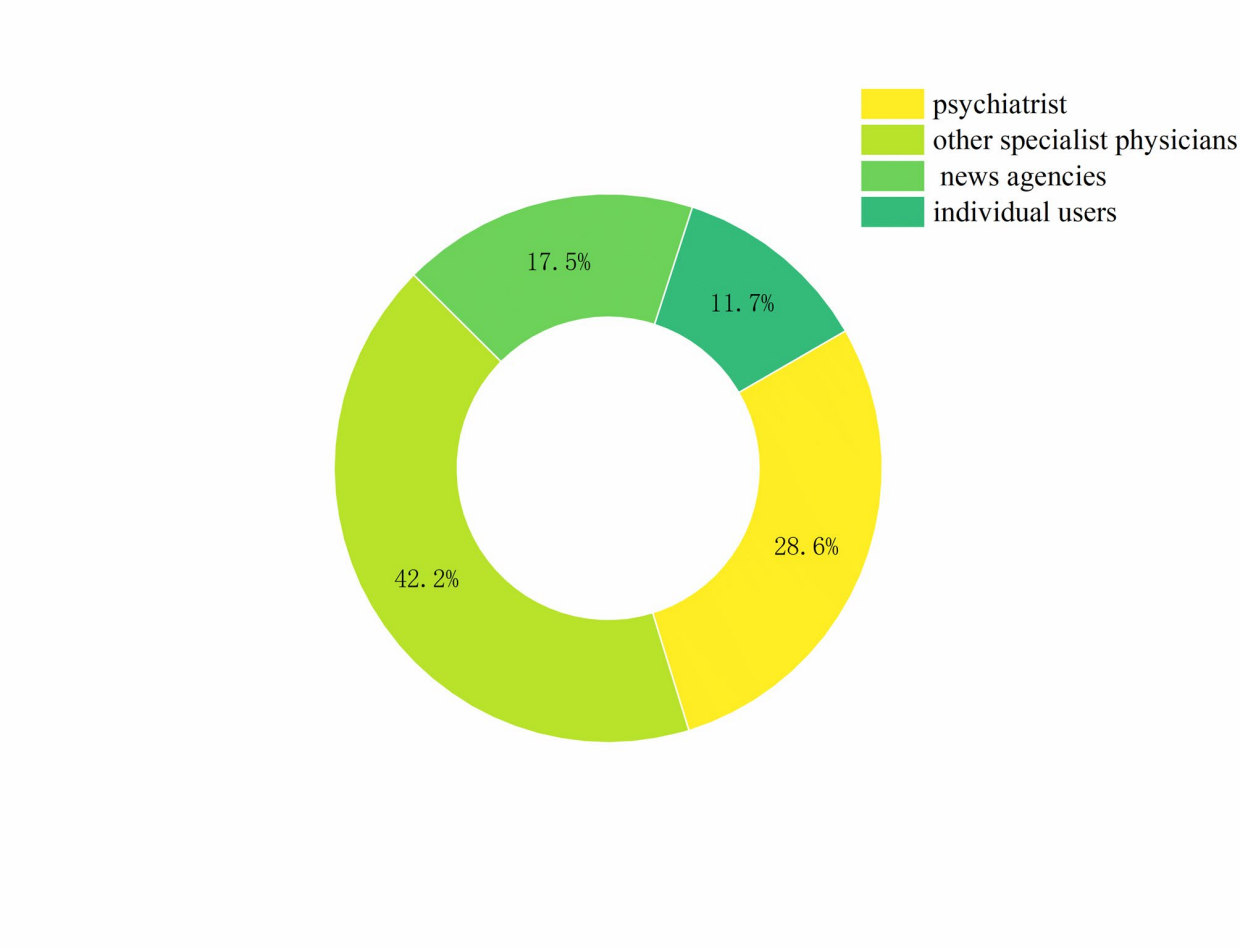
Video source of the total.

The characteristics of the included short videos are summarized in Table 1. Regarding content creators, videos uploaded by healthcare professionals constituted the majority, distributed among psychiatrists, other specialist physicians, news media, and individual users (Fig. 2).

Overall, these videos demonstrated moderate levels of audience engagement. The median counts for likes, comments, favorites, and shares per video were 447 (IQR 66-3967.75), 31 (IQR 5-249.75), 122 (IQR 19-1405), and 129 (IQR 13.75-1496.75), respectively. The median video duration was 41 s (IQR 24-86.25). In terms of quality assessment, the median scores were 2 (IQR 2–3) for the GQS, 2 (IQR 1–3) for the JAMA benchmark, and 2 (IQR 2–3) for the mDISCERN instrument.

### Short videos content analysis

**Fig. 3:**
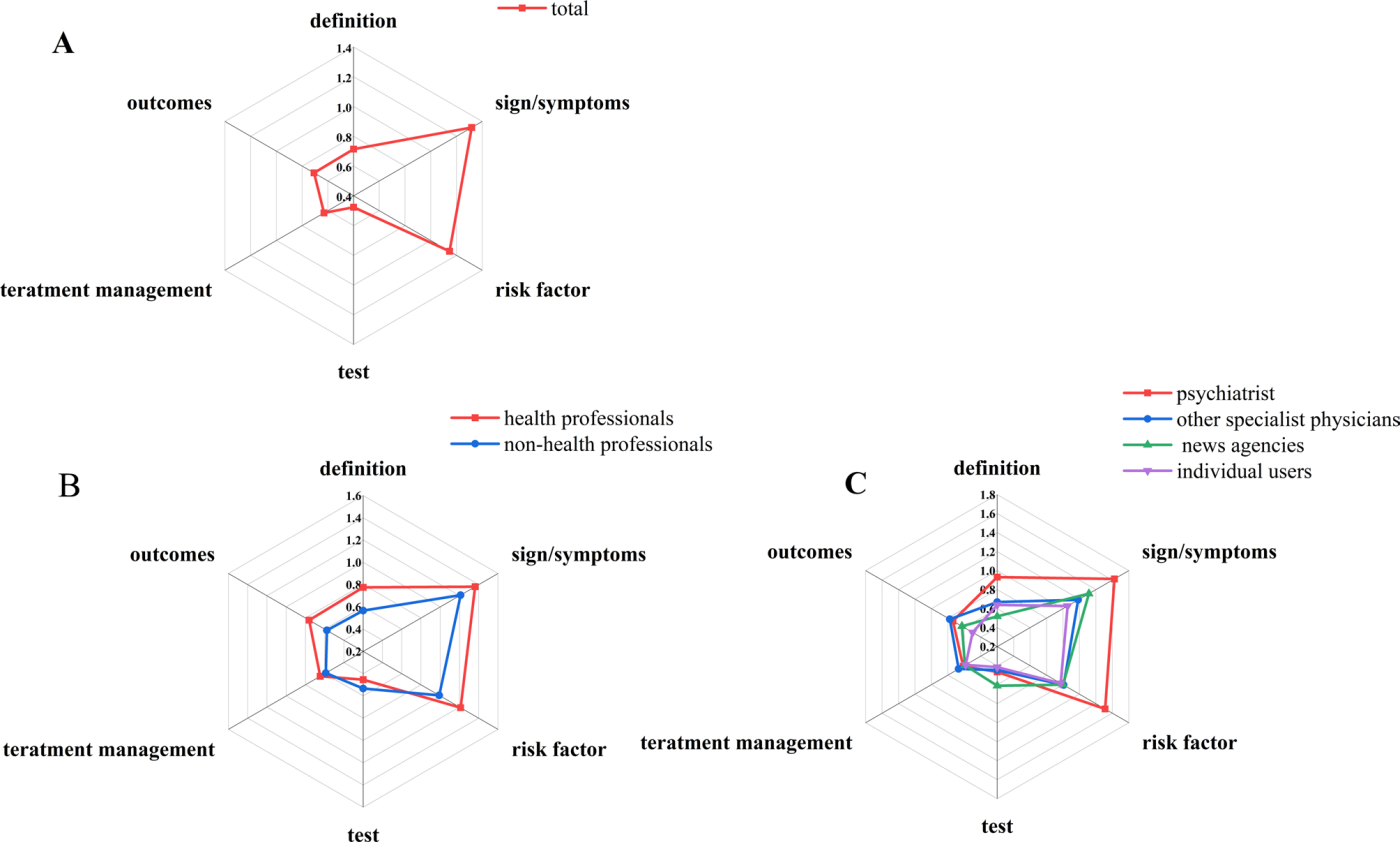
The overall content comprehensiveness of videos in this study (A) and comparisons of content comprehensiveness (B) between health professionals and non-health professionals; (C) among psychiatrists, other specialist professionals, news agencies and individual users.

A structured approach was employed to evaluate video materials based on six core methodological domains. As presented in Table 2, few videos provided comprehensive information on sleep disorders. Specifically, 34.4% of videos lacked any content on detection, while 40.3% only minimally addressed treatment details. A minimal coverage of the definition of sleep disorders was found in 37.7% of the sample, and outcomes were either not mentioned or scarcely covered in 52.6% of videos. In contrast, a greater proportion of videos contained substantive information on the specific symptoms and risk factors of sleep disorders, with 35.1% and 33.1% providing detailed coverage in these respective areas (Fig. 3).

### Videos quality and reliability assessments

As shown in Table 3, group comparisons based on video sources revealed significant differences. Video duration demonstrated a statistically significant difference across groups (*p* < 0.001, Kruskal-Wallis H test), with videos from individual users being the longest (median 117.5 s), followed by those from other specialist physicians. In contrast, no statistically significant differences were observed among the four source groups regarding engagement metrics, including likes (*p* = 0.308), comments (*p* = 0.074), favorites (*p* = 0.459), and shares (*p* = 0.185). Furthermore, videos created by psychiatrists and other specialists received notably higher scores in quality and reliability, whereas those from news agencies received the lowest. Through two different grouping methods, we compared the scores of GQS, mDISCERN, and JAMA respectively.

**Fig. 4:**
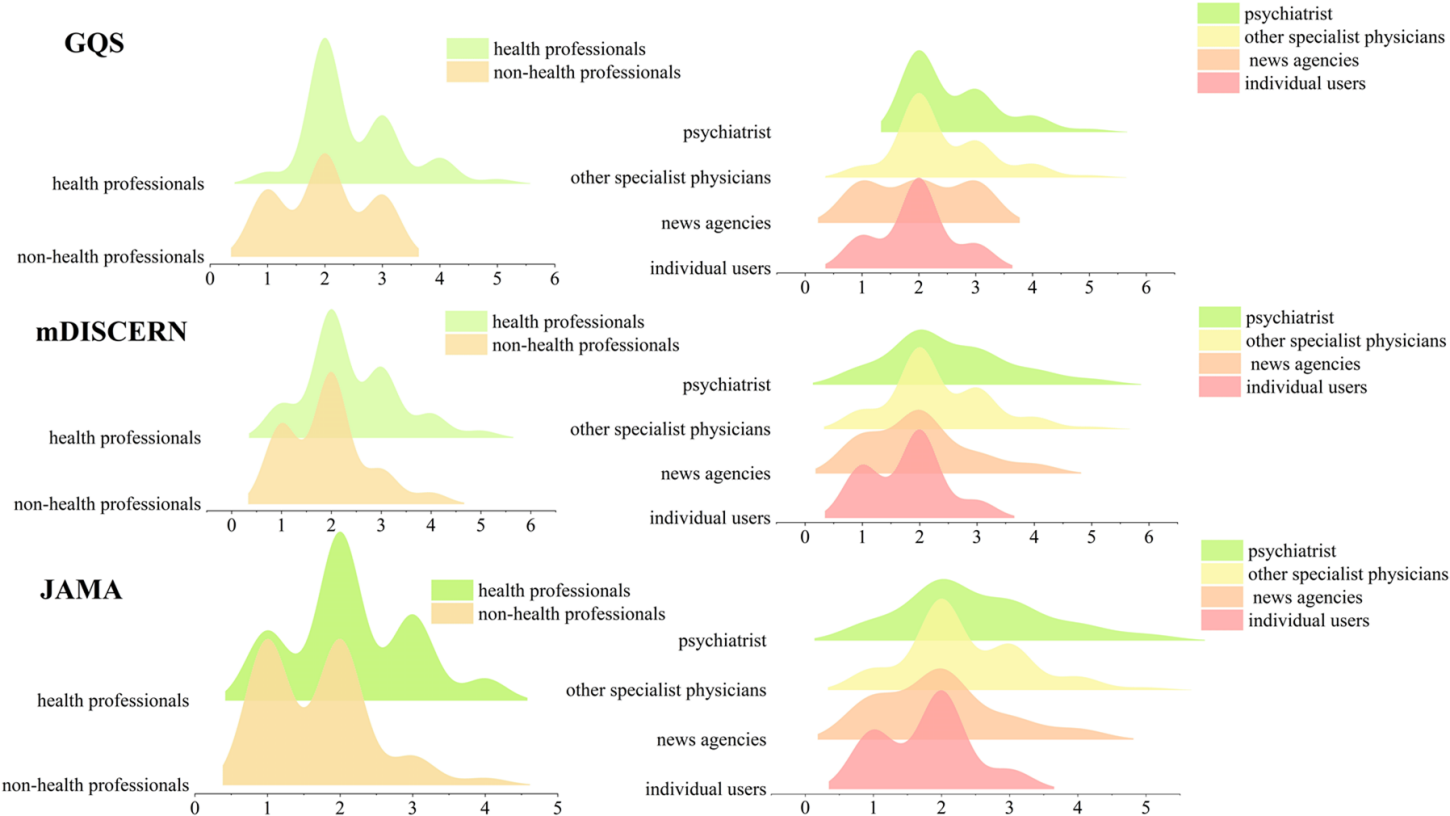
Comparison of video quality and reliability assessment scores.

To thoroughly analyze these differences, we evaluated the quality metrics using two separate grouping paradigms: a detailed four-group source comparison (reported in Table 3) and a consolidated two-group comparison comparing healthcare professionals against non-healthcare professionals (illustrated in Fig. 4). This approach allows us to examine both specific creator behaviors and broader professional versus layperson trends.

### Correlation analysis

**Fig. 5:**
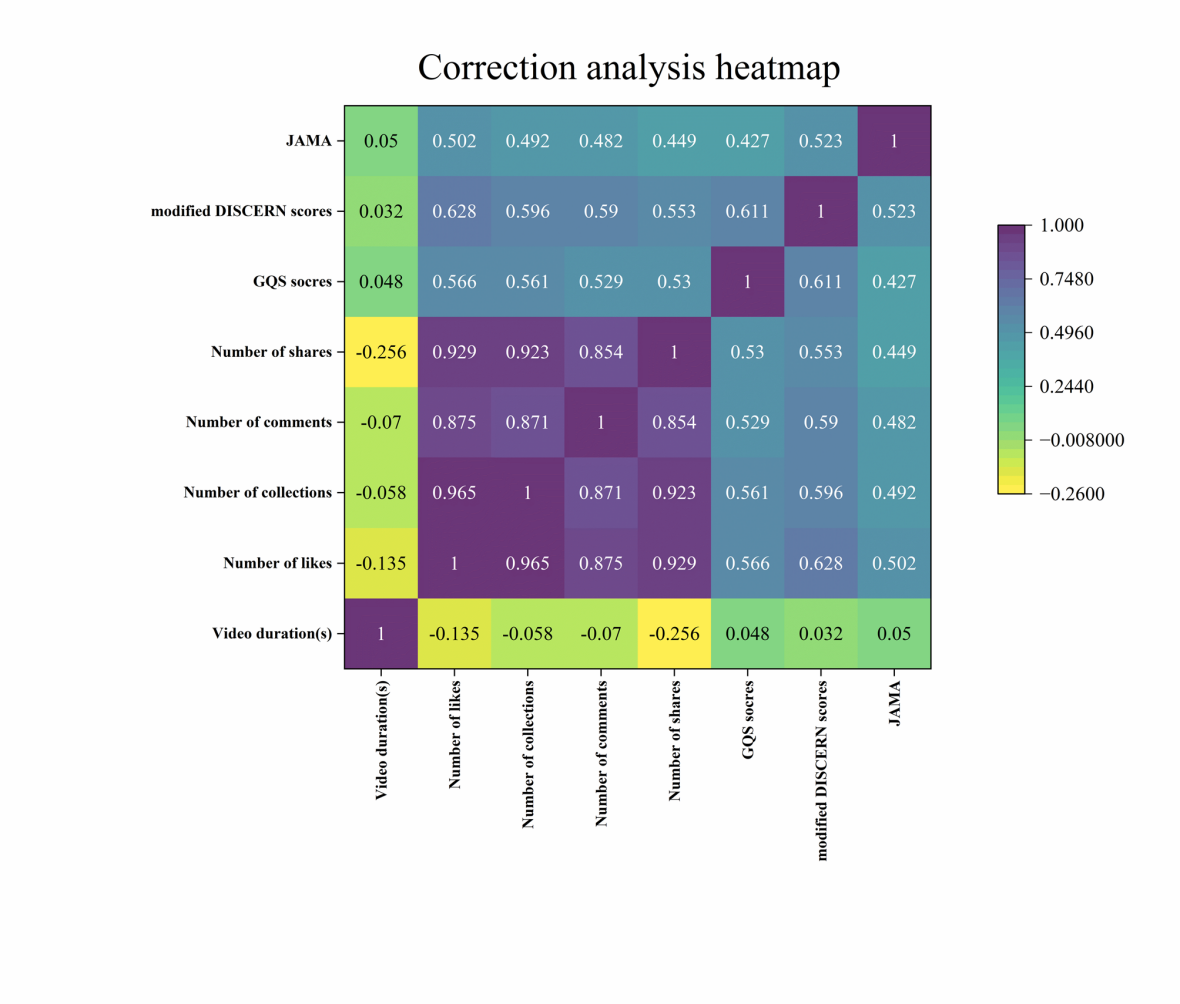
Correlation heatmap between video metrics and quality scores.

Analysis revealed significant associations between engagement indicators and quality scores. Specifically, the Global Quality Scale (GQS) showed positive correlations with all measured interaction metrics (likes, comments, favorites, shares). Furthermore, GQS scores were interrelated with both mDISCERN and JAMA assessment results. Similarly, the mDISCERN score showed significant correlations with all four engagement metrics (likes, comments, favorites, shares), the GQS score, and the JAMA score. Correspondingly, the JAMA score was also correlated with all engagement metrics and the other two quality scores. Notably, video duration did not demonstrate a significant correlation with any of the quality measures (JAMA, *p* = 0.531; mDISCERN, *p* = 0.688; GQS, *p* = 0.553) (Fig. 5).

**Table 3:** Comparison of different video source.

	Psychiatrist	Other specialist physicians	News agencies	Individual users	*p*
Video duration [s, median (IQR)]	29 (25.25–80.75)	44 (30–73)	10 (8–28)	117.5 (84.75–253)	< 0.001
Number of likes [median (IQR)]	1023.5 (103–6583)	287 (49-2497)	561 (97-3916)	472 (55.5–3575)	0.308
Number of collections [median (IQR)]	166 (24.75–1695)	68 (13-975.5)	115 (12-1170)	223 (22.75-1868.25)	0.459
Number of comments [median (IQR)]	74 (14.25–544)	23 (4-117)	18 (2-195)	16.5 (4.5-1192.25)	0.074
Number of shares [median (IQR)]	190.5 (25-2502.75)	73 (11-646.5)	284 (23-1623)	94 (10.25–1502)	0.185
GQS scores [median (IQR)]	2 (2–3)	2 (2–3)	2 (1–3)	2 (1.75-2)	0.009
modified DISCERN scores [median (IQR)]	2 (2–3)	2 (2–3)	2 (1–2)	2 (1–2)	0.012
JAMA scores [median (IQR)]	2 (2–3)	2 (2–3)	1 (1–2)	2 (1–2)	0.004

GQS: Global Quality Score; IQR: interquartile range.

## Discussion

### Principal findings and quality distribution

This cross-sectional study systematically evaluated the quality, reliability, and content coverage of sleep disorder-related short videos on Douyin. The findings indicate that the source of the video significantly influences content quality. Videos created by certified healthcare professionals—particularly psychiatrists and other specialist physicians—received consistently higher scores on the GQS, modified DISCERN, and JAMA instruments compared to non-professional sources. This aligns with existing health communication literature emphasizing the critical role of professional expertise in ensuring the scientific accuracy and reliability of health-related social media content^18^.

Nonetheless, the overall clinical quality of the assessed video sample was moderate, with median scores of 2 across all three standardized instruments. A substantial proportion of the videos omitted or minimally addressed crucial clinical parameters, such as detection/diagnosis (34.4%), treatment details (40.3%), and prognosis or clinical outcomes (52.6%). While these omissions represent clinical informational gaps, we must acknowledge the inherent “completeness paradox” of short-form media. With a median video duration of only 41 s in our sample, it is practically impossible for creators to deliver highly detailed, comprehensive medical lectures covering etiology, diagnostic tests, multi-line treatments, and long-term prognoses. Therefore, these content gaps should not be interpreted simply as a failure of individual creators, but rather as a reflection of the medium’s physical formats and constraints. The absence of mandatory peer-review systems or platform-led clinical fact-checking mechanisms further contributes to these structural content limitations on short-video apps^11^. Consequently, the primary value of short videos on Douyin lies in initial public health outreach, symptom recognition, and destigmatization, rather than structured, comprehensive clinical education.

### The complementary value of non-professional content

While healthcare professionals scored significantly higher on standardized quality instruments, non-professional creators, including individual users, play an important and complementary role in the digital health ecosystem. Although their content often relies on anecdotal or personal experiences rather than clinical, evidence-based data, these videos provide valuable peer support, normalize conversations around sleep struggles, and help reduce the intense social isolation frequently experienced by patients with chronic sleep disorders. Rather than dismissing layperson content entirely, future public health initiatives should focus on guiding individual users to share their lived experiences safely without dispensing unverified clinical advice.

### Qualitative observations on unverified claims

Our qualitative observations during the coding process revealed that several videos promoted unverified treatments, including certain Traditional Chinese Medicine therapies and oversimplified lifestyle interventions, without referencing established clinical guidelines. Such contents have the potential to mislead viewers and delay appropriate medical care^5^. In addition, although specific symptoms like difficulty falling asleep were widely described, critical contextual information regarding formal diagnosis or clear indications for seeking professional help was frequently absent, potentially increasing the risk of self-misdiagnosis or unnecessary health anxiety among viewers^19^.

These omissions can lead to a risk of self-diagnosis and self-treatment by viewers^20^. Even among professional creators, occasional promotions of unvalidated treatments inconsistent with established guidelines were observed, highlighting the ongoing need for improved self-regulation and strict adherence to professional clinical standards^21^.

However, because misinformation prevalence was not mathematically operationalized or statistically modeled as a primary outcome in this study, these findings should be interpreted cautiously as qualitative observations of unverified claims rather than a systemic, measured prevalence rate. It is important to clarify that these observations, while salient and worthy of discussion, were not derived from a formal qualitative content analysis. They are presented here as noteworthy themes identified during data engagement, meriting systematic investigation in future research^17^.

### Causality vs. correlation in user engagement

Our findings showed a positive correlation between clinical quality scores (GQS, mDISCERN, and JAMA) and user engagement metrics. However, we must caution against implying a causal relationship. High clinical quality does not directly cause high user engagement. Instead, this correlation is likely mediated by other confounding platform dynamics, such as the engaging presentation skills of qualified medical professionals, platform algorithms that favor authoritative sources, or the pre-existing follower base of verified creators.

### Methodological limitations and visual quality assessment

This study has several limitations. First, the analysis was restricted to Douyin, and the results may not generalize to other platforms or cultural contexts^22^. Second, due to the rapidly evolving nature of social media content, the findings reflect a specific timeframe and may not capture recent trends. Third, although evaluators were trained and a double-blinded process was used, subjective judgment in quality assessment remains possible^23^. Fourth, correlations observed between engagement metrics and quality scores in our study are observational and do not imply causation; Fifth, the impact of posting times on engagement metrics was not mathematically adjusted, which might affect the interaction analysis. Sixth, the sample size of this study is small, and the distribution of creators is uneven, which may introduce selection bias.

Seventh, traditional quality assessment tools (DISCERN, JAMA, GQS) were originally developed for written consumer health information and may not fully capture short-form video characteristics. They do not account for the rich, multimodal characteristics of short-form video. Modern research in digital media indicates that user experience, attention, and comprehension are heavily shaped by visual and auditory elements, including graphic quality, pacing, text overlays, and audio-visual correspondence. Advanced quality assessment models—such as blind image quality estimation via distortion aggravation^24^, blind quality assessment based on pseudo-reference images^25^, and unified blind quality evaluation of compressed screen content^26^—highlight how technical rendering, compression artifacts, and visual noise affect information digestion. Similarly, subjective quality assessment in the wild^27^ and objective evaluation of enhanced visual frames^28^ play vital roles in shaping viewer perception.

Additionally, our current study evaluated content primarily through clinical and textual templates, which may overlook critical auditory and multimodal elements. Short-form video is inherently multimodal, and the overall user experience is heavily shaped by both visual and auditory information. Studies on audio-visual signals highlight that subjective and objective quality depend on the seamless integration of sound and picture^29^. For instance, a multimodal saliency model for videos with high audio-visual correspondence demonstrates that strong alignment between the visual presentation and spoken voice significantly enhances visual attention and cognitive processing^30^. Moreover, recent advancements in quality assessment for AI-generated media using instruction tuning^31^. Suggest that future research could leverage automated, multimodal models to dynamically evaluate both technical production and clinical content alignment on social media. Future studies should include cross-platform comparisons and explore automated, AI-assisted tools for more scalable and objective content evaluation^32^.

Finally, our search strategy targeted the ‘top’ videos returned by Douyin’s default search ranking algorithm. While this approach captures the videos with the highest reach and cumulative visibility, it may introduce algorithmic selection bias. Douyin’s search ranking algorithm heavily prioritizes user engagement and virality. Therefore, our sample of ‘top’ videos likely skews toward highly popular, potentially commercialized, or sensationalized content, which may not accurately reflect the organic personalized feed or echo chambers experienced by a newly registered or typical user searching for sleep-related information.

## Conclusion

Our study systematically evaluated the quality of sleep disorder–related short videos on Douyin (the Chinese version of TikTok). The findings revealed moderate overall quality (median scores: GQS = 2, JAMA = 2, mDISCERN = 2), suggesting the need for greater efforts to standardize health-related content on such platforms. Furthermore, videos created by healthcare professionals demonstrated higher quality. In addition, video quality scores were positively correlated with influence metrics, implying that content creators could potentially enhance their impact by improving video quality. This study serves as a preliminary exploration with several limitations. Subsequent research should validate these findings across diverse social media environments and analyze audience engagement patterns to optimize digital health communication.

## Supplementary Information

Below is the link to the electronic supplementary material.

Supplementary Material 1

Supplementary Material 2

Supplementary Material 3

## Data Availability

The original video data supporting the findings of this study are publicly available on Douyin (https://www.douyin.com/). The datasets generated and analyzed during the current study (e.g., assessment scores, engagement metrics) are available from the corresponding author, Lina Wang, upon reasonable request.
