# Supplementary material for: A cross-sectional assessment of the quality and reliability of sleep disorder information on Douyin (TikTok in China)
Source: Sci Rep. 2026 Jun 3;16:25389. doi: 10.1038/s41598-026-54965-4 (PMC13473567; doi:10.1038/s41598-026-54965-4)
Supplement: Supplementary file 1 [file 41598_2026_54965_MOESM1_ESM.docx]

**Supplementary Table 2: Global Quality Score (GQS) Scale (Scoring from 1 to 5)**

| **GQS Definition** | **Score** |
| --- | --- |
| Lacks clarity and coherence, with significant information gaps, offering minimal value to patients | 1 |
| Substandard in clarity and structure, includes some details but omits critical topics, limiting its utility for patients | 2 |
| Moderate quality, with key information sufficiently covered | 3 |
| Highly informative with smooth progression, covers most relevant information, valuable for patients | 4 |
| Outstanding in both content and delivery, exceptionally beneficial for patients | 5 |
