# Supplementary material for: A cross-sectional assessment of the quality and reliability of sleep disorder information on Douyin (TikTok in China)
Source: Sci Rep. 2026 Jun 3;16:25389. doi: 10.1038/s41598-026-54965-4 (PMC13473567; doi:10.1038/s41598-026-54965-4)
Supplement: Supplementary file 2 [file 41598_2026_54965_MOESM2_ESM.docx]

Table S1. The Journal of the American Medical Association (JAMA) benchmark criteria.

| Score* | Score component | |
| --- | --- | --- |
| 1 score | Authorship | Author and contributor credentials and their affiliations should be provided. |
| 1 score | Attribution | Clearly lists all copyright information and states references and sources for content. |
| 1 score | Currency | Initial date of posted content and subsequent updates to content should be provided. |
| 1 score | Disclosure | Conflicts of interest, funding, sponsorship, advertising, support, and video ownership  should be fully disclosed. |

*The criteria of each aspect were scored separately, and 1 point for each criterion with a total score of 4 points.
