# Supplementary material for: A cross-sectional assessment of the quality and reliability of sleep disorder information on Douyin (TikTok in China)
Source: Sci Rep. 2026 Jun 3;16:25389. doi: 10.1038/s41598-026-54965-4 (PMC13473567; doi:10.1038/s41598-026-54965-4)
Supplement: Supplementary file 3 [file 41598_2026_54965_MOESM3_ESM.docx]

**Supplementary Table 1: Modified DISCERN quality assessment criteria for evaluating video reliability. (Award 1 point for a 'yes' response, 0 points for a 'no' response)**

**Reliability Score**

1. Does the video convey information in a clear, succinct, and easily comprehensible manner?

2. Are references to credible sources provided?

3. Is the material presented impartially and without favoritism?

4. Are supplementary content resources indicated for further patient exploration?

5. Are any uncertain aspects of the content acknowledged?
